# Association Work in Virginia and Chicago

**Published:** 1897-02

**Authors:** 


It is refreshing to read the history of such association work which has been accomplished in Virginia, as is recorded in this number of the Journal. Equally well may it be said that no more unattractive field existed than that of the "Old Dominion". The faithful, earnest, and successful work of the members of this Association deserves the highest commendation, and the entire profession owes them a debt of gratitude for their earnest, persistent labors.

---

Signs of the times are not wanting to impress upon our members a realization of the duties of the hour for veterinarians to accept. Association work grows stronger and more forceful as the needs become more pressing, and the recent formation of an active association in Chicago bids well to aid every movement looking to a betterment of our field of work. It is doubly fortunate for a society to find a good secretary, on whose shoulders falls so much of the labor that measures the success of every organization. The "Windy City" Society seems specially favored in this direction, and the Journal gladly extends its congratulations with a full measure of appreciation of the worth of such an officer.

